# Dr. David Cerna

**Published:** 1893-01

**Authors:** 


### BIOGRAPHICAL.

### DR. DAVID CERNA,

The new Medical Teacher in Texas Medical College, who has recently been appointed Demonstrator of Physiology in the Medical Department of the University of Texas, was born in San Buenaventura, State of Coahuila, Mexico; received his elementary education at his native town. At the age of 14, he was sent to Philadelphia, where his first scholastic year was spent in acquiring a knowledge of the English language, which he thoroughly understands and speaks almost without any foreign accent. At the end of the second year in La Salle College, the young Mexican obtained the second place in his class and won a silver medal. In the third year, having already completely mastered the language, he carried off the first prize, a gold medal.

After leaving La Salle College, Dr. Cerna entered the Medical Department of the University of Pennsylvania, in 1874, from which institution he received his medical degree, in March, 1879, graduating with high honors. He was awarded one of the alumni prizes for his thesis entitled "Thevetia Iccotli and Its Glucoside." In June of the same year, after a most rigid examination, he was among the privileged few who received from the same University, the degree of Doctor of Philosophy, and received the "George B. Wood" prize for his essay entitled "Phenic Acid: Its Poisonous Effects and the Soluble Sulphates as Antidotes."

In 1890, Dr. Cerna returned to his native country, and engaged in the practice of his profession. He returned, in 1889, to Philadelphia, and devoted himself to the science of medicine. He shortly after became connected with the University of Pennsylvania as assistant in the laboratory of experimental therapeutics. He then became assistant in physiology, and more recently was appointed demonstrator of, and lecturer on, experimental therapeutics in the same institution, a position that he resigned to come to the University of Texas.

In 1890, Dr. Cerna carried off the prize offered by the Medical Society of the county of New York, for his work entitled "A Physiologic and Therapeutic Study of Hydrastis Canadensis." In the following year he received from abroad a diploma that made him a corresponding fellow of the Sociedad Española de

Higiene, of Madrid, and this year was duly elected Fellow of the College of Physicians of Philadelphia. He is also a member of the Philadelphia County Society, and of the Pathological Society of Philadelphia.

Dr. Cerna has been an active contributor to medical literature. His publications, which number a good many, have chiefly contained the results of original investigation. He is one of the associate editors of Sajous' *Annual of the Universal Medical Sciences*, and has just finished a little book entitled "Notes on the Newer Remedies."

The University of Texas has made a judicious selection in the appointment of Dr. Cerna upon its staff of medical teachers, and is to be congratulated.

It is here announced that Dr. Cerna will be a regular contributor to the JOURNAL, and will have charge of and edit the DEPARTMENT OF THERAPEUTICS.

